# PCR ribotypes of *Clostridioides difficile* across Texas from 2011 to 2018 including emergence of ribotype 255

**DOI:** 10.1080/22221751.2020.1721335

**Published:** 2020-02-10

**Authors:** Anne J. Gonzales-Luna, Travis J. Carlson, Kierra M. Dotson, Kelley Poblete, Gabriela Costa, Julie Miranda, Chris Lancaster, Seth T. Walk, Shawn Tupy, Khurshida Begum, M. Jahangir Alam, Kevin W. Garey

**Affiliations:** a Department of Pharmacy Practice and Translational Research, University of Houston College of Pharmacy, Houston, TX, USA; b Department of Clinical Sciences, Fred Wilson School of Pharmacy, High Point University, High Point, NC, USA; c Division of Clinical and Administrative Science, Xavier University of Louisiana College of Pharmacy, New Orleans, LA, USA; d Department of Microbiology & Immunology, Montana State University, Bozeman, MO, USA; e Texas Department of State Health Services, Austin, TX, USA

**Keywords:** *Clostridium difficile*, ribotype, epidemiology, active surveillance, emerging strains

## Abstract

*Clostridioides difficile* infection (CDI) is the most prevalent healthcare-associated infection in the United States and carries a significant healthcare system burden. As part of an ongoing, active surveillance system of *C. difficile* throughout Texas, the objective of this study was to assess changes in *C. difficile* ribotypes of clinical isolates obtained from hospitalized patients in Texas over the past seven years. Fifty hospitals located in Texas, USA sent *C*. *difficile* positive stool specimens to a centralized laboratory for PCR ribotyping and toxin characterization between 2011 and 2018. Data collected included specimen collection date, patient age, and sex. Strain genotypes were compiled, and changes in ribotype distribution over time were assessed. Overall, 7796 samples were ribotyped from predominately female patients (58.4%) aged 62 ± 19 years. Samples were obtained from all geographic regions of Texas including Houston/Southwest region (*n* = 5129; 85%), Dallas/North Texas (*n* = 579, 9.6%), Central Texas (*n* = 164; 2.7%), and South Texas (*n* = 162; 2.6%). The 10 most common ribotypes comprised 73% of all isolates tested during the study period. The most common ribotypes were 027 (17.5%), followed by 014–020 (16.1%), 106 (11.6%), and 002 (9.1%). The prevalence of ribotypes 027, 001, and 078–126 declined significantly over time, while ribotypes 106 and 054 increased in prevalence (*P* < 0.001). Furthermore, the emergence of a novel ribotype 255 strain was observed. Differences in ribotype distribution were also noted based on age and geographic distribution (*P* < 0.001, each). This seven-year study demonstrated changing molecular epidemiology of *C. difficile* in Texas, including the emergence of a novel ribotype 255.

## Background

*Clostridioides difficile*, a spore-forming, toxin-producing, anaerobic bacteria, causes gastrointestinal disease with symptoms ranging from mild diarrhea to pseudomembranous colitis [1]. *C. difficile* infection (CDI) is the most prevalent healthcare-associated infection (HAI) in the United States (US) and carries a significant burden on the US healthcare system [2]. Similar to other HAIs, *C. difficile* rates can be reduced with an understanding of the risk factors, strict infection control, and antimicrobial stewardship [3–5]. Importantly, knowledge of and access to local ribotype patterns have been shown to impact the prevalence of epidemic strains, emphasizing the impact surveillance efforts can have [4].

*C. difficile* strain ribotyping using polymerase chain reaction (PCR) is one method used to understand the molecular epidemiology and transmission dynamics of CDI. Previously, *C. difficile* genotyping, including PCR ribotyping, has been used to identify hypervirulent *C. difficile* strains [6]. Furthermore, characteristic phenotypic susceptibility patterns of certain *C. difficile* strains have been used to direct antimicrobial stewardship efforts to prevent their spread [7–9]. Thus, ribotyping surveillance efforts can be used to direct infection control and antimicrobial stewardship efforts.

Ribotyping surveillance data has shown that the molecular epidemiology of the disease is shifting. The prevalence of the epidemic ribotype 027 is decreasing globally, while ribotypes 106 and 017 have become the most common strains in Europe and Asia, respectively [10–15]. The most recent national data provided by the Centers for Disease and Control (CDC) shows similar trends in the US, however, this data is derived from 1000 to 1500 annual samples submitted from ten states, not including Texas [13]. An understanding of local and regional epidemiology has been shown to influence the ribotype distributions in several positive ways. As England was one of the first countries to implement mandatory reporting and centralized ribotyping, it serves as an example for many of the benefits of such a system. After implementing enhanced surveillance in 2007, England saw a decrease in the overall incidence of CDI after years of increases, and has been able to implement targeted antimicrobial stewardship and infection control efforts to decrease the rates of specific ribotypes, and 027 in particular [4,16]. Multiple other surveillance systems have been used to identify novel and emerging ribotypes, [17–19] which in turn allow earlier interventions and infection control efforts.

Unlike countries such as the United Kingdom, the US does not have a nationalized surveillance system of *C. difficile* [20]. As part of a collaboration with the Texas Department of State Health Services, the University of Houston established the *C. difficile* Across Texas United Surveillance (CAcTUS) Network in 2011 to better understand the molecular epidemiology of *C. difficile* throughout Texas. The network is used to prospectively track outbreaks and identify the emergence of new strains. Our surveillance system provides up-to-date reporting to participating institutions, and identified one such outbreak due to a clonal strain in a long-term care facility [21]. Here we describe the changes in *C. difficile* ribotypes obtained from patient samples over the past seven years.

## Methods

### Collection, culture, and typing

The CAcTUS Network is a centralized *C. difficile* surveillance system based in a translational research laboratory at the University of Houston College of Pharmacy in Houston, Texas. Participation in CAcTUS was voluntary and supplies and standardized reporting sheets were provided to interested institutions. Participating institutions sent leftover *C. difficile* diagnostic stool samples on a weekly to monthly basis to our centralized lab for *C. difficile* growth and PCR-ribotyping. Samples included in this report were collected between 1 January 2011 and 31 December 2018. Collection of stools for *C. difficile* testing was conducted as part of routine clinical care as per individual hospital algorithms. Outbreaks were reported back to and investigated by individual institutions at the time of identification.

*C. difficile* culture and ribotyping were conducted at the CAcTUS centralized lab. Samples were plated onto selective cefoxitin-cycloserine-fructose agar (CCFA) plates (Anaerobe Systems, Morgan Hill, CA) and anaerobically incubated for 48–72 h for culture. Characteristic *C. difficile* colonies were tested using latex agglutination reagent (Oxoid, Hampshire, England) and presence of triose phosphate isomerase and toxin genes was determined using multiplex PCR [22]. Fluorescent PCR ribotyping was performed as previously described [23]. This technique does not distinguish between all ribotypes; therefore, some ribotypes are reported as combined (e.g. 053–163, and 014–020). Maintenance of the library to match ribotypes with an international collection is completed by Montana State University (https://thewalklab.com/tools/).

### Clinical data

Information requested of all participating sites included *C. difficile* diagnostic testing method (e.g. nucleic acid amplification test (NAAT) or enzyme immunoassay (EIA)), sample collection date, patient hospitalization admission and discharge dates, patient age, and patient sex. Additionally, investigators had full access to the electronic medical records at two large healthcare systems in Houston comprising 13 of the participating hospitals. Hospital-onset CDI (HO-CDI) cases were defined by the CDC multidrug-resistant organism and CDI module [24]. All others were classified as community onset. This study was approved by the University of Houston Committee for the Protection of Human Subjects (CPHS00128).

### Evaluation of emerging ribotypes

To assess the virulence of emerging strains vs. endemic strains, the clinical outcomes associated with any emerging strain were retrospectively evaluated once identified as an emergent strain in the region. A convenience sample of 50 patients infected with an emerging ribotype was compared to a random sample of 50 patients infected with each ribotype 027 and 014–020, two other endemic ribotypes in the area, over the same time frame (2016–18). The primary outcome was disease severity as classified by the Infectious Diseases Society of America (IDSA) guidelines published in 2017 [3]. Disease severity (mild vs. non-mild) was compared between any emerging ribotype and ribotypes 027 and 014–020 controlling for age, Charlson Comorbidity score, and serum albumin using multivariable logistic regression. Other outcomes assessed include initial clinical cure, defined as the absence of symptoms and/or treatment failure on day 7 of treatment, 30- and 90- day recurrence, complicated disease, and all-cause 30-day mortality.

### Statistical analysis

To be able to compare regional diversity of ribotypes, Texas was divided into five geographic regions for analysis derived from the Texas Department of State Health Services (https://www.dshs.texas.gov/regions/). The regions included Central Texas (Austin and surrounding areas), North Texas (including Dallas-Fort Worth, Lubbock), South Texas (San Antonio, Rio Grande Valley), and the Gulf Coast (greater Houston area, Corpus Christi). Changes in ribotype distribution over time were assessed by linear regression. Differences in age, gender, and other clinical data was compared using Pearson’s chi-squared test (categorical data) or Student’s T-test (continuous variables). All *p*-values were from 2-sided tests, and results were deemed statistically significant at *p* < .05. All statistical analyses were performed using STATA, version 15.1 (StataCorp LLC, College Station, TX) or SPSS, version 25.0.0.0 (IBM Corp., Armonk, NY).

## Results

### Geography and patient characteristics

**Figure 1.**
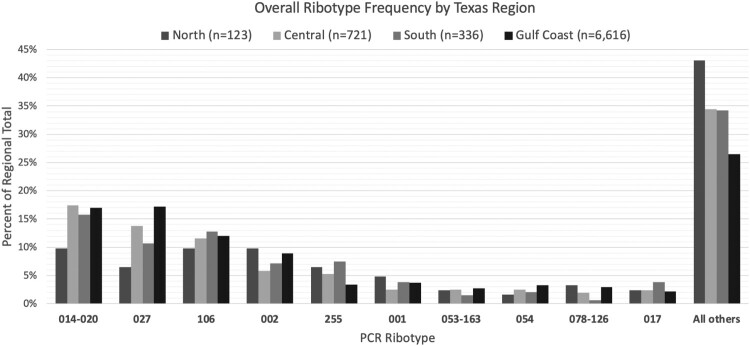
Ribotype Frequency by Texas Region, 2011–2018. Regions included Central Texas (Austin and surrounding areas), North Texas (including Dallas-Fort Worth, Lubbock), South Texas (San Antonio, Rio Grande Valley), and the Gulf Coast (greater Houston area, Corpus Christi). Abbreviations: PCR, polymerase chain reaction.

A total of 7796 unique isolates were included from 50 hospitals between 2011 and 2018. The complete count of total samples received began in 2018, of which 1653 of 2517 isolates (66%) were successfully ribotyped that year. The average bed size of hospitals submitting samples was 352 with a wide range of bed size (<100 beds [small]: 16%; 100–250 beds [medium]: 28%; 250–500 beds [large]: 32%, or >500 beds [very large]: 24%). The majority of the samples were submitted from hospitals within the Gulf Coast region (84.8%), followed by the Central region (9.2%) (Figure 1). No samples were submitted from hospitals in the West Texas region. The number of samples included from each site varied over the time frame and ranged from 2 to 596 samples per hospital. Demographic data was provided for 5165 (66%) of samples. Of that cohort, the majority were female (58.4%) with a mean age of 62 ± 19 years.

### Diagnostics

The diagnostic tests used varied over the study time frame, however, 38 of the hospitals utilized PCR testing alone at the time of sample submission, representing 80.7% of the samples included (*n* = 6295 samples). Another five hospitals utilized a glucose dehydrogenase (GDH) screen + EIA combination (6.2%, 480 samples), one used PCR + EIA combination (7.2%, 562 samples), one utilized GDH + PCR combination (2.6%, 206 samples), and five sites did not report the diagnostic testing used (3.2%, 253 samples).

### Overall ribotype distribution

The most common ribotypes isolated were 014–020 (16.9%), followed by 027 (16.5%) and 106 (12%) (Table 1). The diversity of ribotypes increased over the study time frame; 27 ribotypes were identified in 2011 while 73 were identified in 2018. Furthermore, 82.4% of ribotyped isolates in 2011 belonged one of the 10 most common ribotypes versus 68.3% of 2018 isolates. The largest change in ribotype distribution over time affected ribotype 027, which decreased by 50% from its peak prevalence in 2013. Others that declined significantly were ribotypes 001, 078–126, 053–163, and 017, while ribotypes 106, 054, and 255 increased (*p* < 0.002 for all).

**Table 1.** Annual distribution of ribotypes in Texas.

Ribotype (*N* = 7796)	Year								Percentage change**^†^**	*p*-value
	2011** ***n* = 330	2012** ***n* = 416	2013** ***n* = 570	2014** ***n* = 166	2015** ***n* = 453	2016** ***n* = 1997	2017** ***n* = 2211	2018** ***n* = 1653		
014–020** ***n* = 1317	52 (15.8)	65 (15.6)	84 (14.7)	36 (21.7)	75 (16.6)	331 (16.7)	369 (16.7)	305 (18.5)	+1.9	0.092
027** ***n* = 1284	71 (21.5)	108 (26)	158 (27.7)	40 (24.1)	86 (19)	298 (14.9)	299 (13.5)	224 (13.5)	−11.1	**<0.001**
106** ***n* = 935	34 (10.3)	37 (8.9)	65 (11.4)	17 (10.2)	50 (11)	203 (10.2)	315 (14.2)	214 (12.9)	+3.6	**0.001**
002** ***n* = 671	28 (8.5)	33 (7.9)	55 (9.6)	19 (11.4)	44 (9.7)	178 (8.9)	188 (8.5)	126 (7.6)	−1.3	0.27
255** ***n* = 293	1 (0.3)	0 (0)	0(0)	1 (0.6)	3 (0.7)	91 (4.6)	108 (4.9)	89 (5.4)	+9.9	**<0.001**
001** ***n* = 281	25 (7.6)	26 (6.3)	21 (3.7)	6 (3.6)	26 (5.7)	67 (3.4)	75 (3.4)	35 (2.1)	−6.1	**<0.001**
054** ***n* = 247	9 (2.7)	17 (4.1)	15 (2.6)	9 (5.4)	22 (4.9)	54 (2.7)	54 (2.4)	67 (4.1)	0	0.98
078–126** ***n* = 218	19 (5.8)	21 (5)	42 (7.4)	9 (5.4)	28 (6.2)	45 (2.3)	30 (1.4)	24 (1.5)	−10.5	**<0.001**
053–163** ***n* = 204	19 (5.8)	22 (5.3)	26 (4.6)	7 (4.2)	18 (4)	50 (2.5)	37 (1.7)	25 (1.5)	−8.1	**<0.001**
017** ***n* = 176	14 (4.2)	22 (5.3)	16 (2.8)	3 (1.8)	12 (2.6)	48 (2.4)	41 (1.9)	20 (1.2)	−6	**<0.001**
All others** ***n* = 2170	58 (17.6)	65 (15.6)	88 (15.4)	19 (11.4)	89 (19.6)	632 (31.6)	695 (31.4)	524 (31.7)	+12.9	**<0.001**

Notes: Values displayed as no. (% of annual total). Statistically significant *p* values were remarked in bold.

^†^Calculated from line of best fit.

The overall ribotype distribution differed between the four regions of Texas included (*p* < 0.001) (Figure 1). Ribotype distribution did not differ between sexes (*p* = 0.152), but did vary by patient age (≥65 vs. <65 years; *p* < 0.001). The most common ribotype seen in those aged < 65 years was ribotype 014–020 (19.2% of all strains), which represented only 15% of strains in those ≥65 years (*p* < 0.001). Furthermore, the prevalence of this ribotype group increased between 2011 and 2018 from 10.8% to 17.4% in those ≥65 years and decreased from 24.6% to 18.5% in those <65 years. Although ribotype 027 was more common overall in patients ≥65 years (21.1% vs 11.4%, *p* < 0.001), the prevalence declined similarly between the age categories.

Of the 3877 samples with epidemiologic classifications provided, 2618 (67.5%) were community-onset and 1259 (32.5%) were hospital-onset (Table 2). There was no difference in ribotype distribution between the two categories (*p* = 0.076).

**Table 2.** Ribotype distribution among community-associated vs. hospital-acquired CDIs, stratified by age <65.

Ribotype	Community-onset, *n* (%)			Hospital-onset, *n* (%)		
	Total^†^ *n* = 2618	Age <65 *n* = 1177	Age ≥65 *n* = 1238	Total^‡^ *n* = 1259	Age <65 *n* = 621	Age ≥65 *n* = 627
014–020	445 (17.0)	235 (20.0)	182 (14.7)	218 (17.3)	110 (17.7)	106 (16.9)
027	427 (16.3)	128 (10.9)	278 (22.5)	200 (15.9)	82 (13.2)	116 (18.5)
106	316 (12.1)	145 (12.3)	137 (11.1)	167 (13.3)	78 (15.6)	86 (13.7)
002	255 (9.7)	120 (10.2)	110 (8.9)	105 (8.3)	47 (7.6)	57 (9.1)
255	108 (4.1)	54 (4.6)	51 (4.1)	41 (3.3)	27 (4.3)	14 (2.2)
001	86 (3.3)	43 (3.7)	35 (2.8)	62 (4.9)	34 (5.5)	27 (4.3)
054	67 (2.6)	29 (2.5)	31 (2.5)	42 (3.3)	25 (4.0)	16 (2.6)
078–126	74 (2.8)	35 (3.0)	38 (3.1)	27 (2.1)	17 (2.7)	10 (15.9)
053–163	52 (2.0)	17 (1.4)	32 (2.6)	35 (2.8)	13 (2.1)	22 (3.5)
017	57 (2.2)	32 (2.7)	19 (1.5)	28 (2.2)	19 (3.1)	9 (1.4)
All others	731 (27.9)	339 (28.8)	325 (26.3)	334 (26.5)	169 (27.2)	164 (26.2)

^†^203 patients missing age information, ^‡^11 missing age information.

### Emerging ribotypes

The fifth most common ribotype identified over the study time frame was 255, which grew from 0% to 0.6% of all isolates between 2011 and 2014 to 5.4% of isolates by 2018. This ribotype was more frequently isolated from hospitals in the South (7.4%), Central (6.5%), and North (5.3%) regions than the Gulf Coast region (3.4%). There was no difference in the distribution of ribotype 255 between male and female sex (4.3% vs 3.9%, *p* = 0.41), those ≥65 and <65 years of age (3.7% vs. 4.7%, *p* = 0.08), or community- and hospital-onset disease (4.1% vs. 3.3%, *p* = 0.38).

Since ribotype 255 was identified as an emerging ribotype, clinical outcomes were retrospectively evaluated for patients infected with ribotype 255 compared to patients infected with ribotypes 027 or 014–020. When compared to patients infected with ribotype 027, patients with ribotype 255 had lower median Charlson Comorbitity Index (CCI) scores and were younger on average than those infected with ribotype 027 (Table 3). *C. difficile* disease severity was similar between those infected with ribotype 255 and ribotype 014–020 (*p* = 0.84). In multivariate analysis, patients infected with ribotype 255 had an 87% relative reduction in the odds of severe disease compared to ribotype 027 after controlling for patient age, CCI score, and serum albumin level (OR, 0.13; 95% CI, 0.037–0.433; *p* = 0.001). No differences were seen in the rates of 30-day mortality, or 30- or 90-day recurrence between the three ribotypes (Table 3).

**Table 3.** Baseline characteristics and outcomes associated with an emergent ribotype 255 compared to two other endemic ribotypes in Houston, Texas.

	Ribotype				
	255 *n* = 50	027 *n* = 50	*p*-value 255 vs 027	014–020 *n* = 50	*p*-value 255 vs 014–020
Age, mean years (±SD)	59.3 (±16.8)	69.3 (±13.6)	**0.001**	61 (±18.3)	0.63
CCI score, median (IQR)	2 (1–3)	3 (2–5)	**0.014**	2 (1–4)	0.34
Initial clinical cure, no. (%)	38 (76)	30 (60)	0.09	38 (76)	Not tested
Severe/fulminant disease, no. (%)	19 (38)	40 (80)	**<0.001**	20 (40)	0.83
CDI complications^†^	5 (10)	14 (28)	**0.02**	9 (18)	0.25
30d recurrence, no. (%)	2 (4)	2 (4)	Not tested	3 (6)	0.64
90d recurrence^‡^, no. (%)	5 (10)	10 (20)	0.17	4 (8)	0.70
All-cause 30d mortality, no (%)	5 (6)	8 (16)	0.37	6 (12)	0.75

Abbreviations: Charlson Comorbidity Index (CCI), standard deviation (SD), *C. difficile* infection (CDI).

^†^Includes ICU admission, colectomy, ileus, and toxic megacolon, ^‡^90-day recurrence includes those with 30-day recurrence.

## Discussion

This is the first report detailing a state-wide effort to better understand the molecular epidemiology of *C. difficile* in Texas. International studies have demonstrated that CDI populations are geographically distinct [11,25], but trends in the molecular epidemiology of *C. difficile* in the United States are not well understood. There are several limitations to the current reporting in the USA including small sample sizes which are often dependent on samples collected for clinical trials, inconsistent reporting from year-to-year, and limited geographic coverage [13,26,27]. Our surveillance demonstrates similar patterns to those seen in the most recent national data, with a few exceptions.

 Strengths of the study include a large sample size being one of the largest *C. difficile* molecular epidemiology studies conducted in the U.S. Furthermore, samples from 50 hospitals are included, and represent a diverse patient population, including two pediatric hospitals, one large cancer centre, and a multitude of academic and community hospitals in both urban and rural settings. All samples submitted were included in this report, minimizing the likelihood of a reporting bias. Accordingly, this surveillance is likely an accurate representation of ribotypes in Texas. Other strengths include use of a centralized laboratory with consistent methodology for ribotyping, and a multi-year evaluation. This study also uses, for the first time, an evaluation method to determine the potential “hypervirulence” of any emerging ribotype.

The most commonly identified ribotype in Texas was the grouping of 014 and 020 ribotypes, regardless of community- or hospital-onset designation. Although this differs from the CDC surveillance, which reports these two ribotypes independently, similar findings have been demonstrated in other North American reports [28–30]. Furthermore, the group of ribotypes 014–020 was identified as the most common across Europe in 2008 [11], and more recent reports able to separate the two ribotypes indicate an increase in 014 may be responsible for the increasing prevalence of the group [17]. We have also demonstrated that the ribotype 014–020 is most prevalent in the community environment [23]. The prevalence of ribotype 027 decreased by more than 50% in Texas from its peak in 2012–13, consistent with other reports from the U.S. [27,29,31]. With the decline of this epidemic strain, there was a corresponding increase both in specific ribotypes, such as 106 and 014–020, as well as in the overall diversity of *C. difficile* ribotypes observed in Texas.

Notably, we identified an emergent ribotype 255 over the study time frame. Ribotype 255 has rarely been isolated [29], and attributes of the strain and associated clinical outcomes are not well described. The complete genome of ribotype 255 has recently been published.[32] Our cohort study indicated disease severity and outcomes similar to those seen with ribotype 014–020, but was limited by sample size. Additionally, ribotype 255 was correlated with much lower odds of severe CDI compared to ribotype 027 [22]. Patients infected with ribotype 027 were older and with more comorbidities compared to those infected with ribotype 255, potentially indicating a bias in who is infected with each ribotype. Our study was unable to demonstrate a difference in sex, age, or location of onset in those infected with ribotype 255. Factors potentially contributing to the rise of this ribotype, including antibiotic resistance patterns and virulence factors, warrant further study.

The differences in the distribution of ribotypes infecting those aged ≥65 years and those <65 years appears to be driven by changes in ribotype 014–020. In addition to ribotype 014–020 being more common in those <65 years, the prevalence decreased throughout the study timeframe while increasing in those ≥65 years. The increase of 014–020 in those ≥65 years seemed to correspond with the declining prevalence of ribotype 027. The same was not seen in those <65, with an increased number of different ribotypes observed in this age group. Studies stratifying ribotype distribution changes by age are lacking, and such patterns are difficult to interpret without more information regarding patients’ exposure to various risk factors.

Interestingly, there was no difference between ribotype frequencies seen in community-onset and hospital-onset CDI. This observation is distinct from previous studies, but may be confounded by undocumented healthcare exposure in those with community-onset disease [31,33]. National data (excluding isolates from Texas) indicate that ribotype 106 is the most common amongst community-associated CDI isolates, while ribotype 027 continues to be the most prevalent amongst hospital-acquired CDI [13]. There was an observed difference in the infecting ribotypes within community- and hospital-onset disease when stratified by age <65, and ribotype 027 replaced ribotype 014–020 as the most common ribotype in those ≥65 regardless of onset location.

This study has limitations including an oversampling from the Gulf Coast region and no samples collected from one distinct geographic area. Samples were submitted voluntarily and were not consistently submitted from the same hospitals over the time frame. Data regarding the number of diagnostic tests ordered and overall CDI incidence for the area were unknown, and would provide useful context for ribotype trends if available. Information regarding the annual CDI prevalence per institution was not available, and incidence rates of CDI were unable to be calculated. Lastly, antibiotic utilization data and stewardship initiatives were not accounted for, and we were therefore unable to hypothesize about forces that may have contributed to changes in the ribotype distribution over time and between regions.

## Conclusion

This seven-year surveillance study demonstrated the changing molecular epidemiology of *C. difficile* in Texas, and identified the emergence of a novel ribotype 255. Although overall patterns were similar to those seen in national U.S. data, ribotype 014–020 was uniquely the most common ribotype seen in Texas, and differences in infecting strains were noted when stratifying our population by age. Evidence provided by this surveillance helps expand our understanding of *C. difficile* epidemiology in the U.S. and emphasizes the importance of continued efforts. Continued efforts toward more wide-spread surveillance in the United States should be emphasized as the rate of CDI continues to increase nationally, despite significant infection control and antimicrobial stewardship efforts [34].
